# Genetics, Breeding and Genetic Engineering to Improve Cottonseed Oil and Protein: A Review

**DOI:** 10.3389/fpls.2022.864850

**Published:** 2022-03-10

**Authors:** Man Wu, Wenfeng Pei, Tom Wedegaertner, Jinfa Zhang, Jiwen Yu

**Affiliations:** ^1^State Key Laboratory of Cotton Biology, Key Laboratory of Cotton Genetic Improvement, Ministry of Agriculture, Institute, Cotton Research of Chinese Academy of Agricultural Sciences, Anyang, China; ^2^Zhengzhou Research Base, State Key Laboratory of Cotton Biology, School of Agricultural Sciences, Zhengzhou University, Zhengzhou, China; ^3^Cotton Incorporated, Cary, NC, United States; ^4^Department of Plant and Environmental Sciences, New Mexico State University, Las Cruces, NM, United States

**Keywords:** seed oil content (SOC), fatty acid, seed protein content (SPC), amino acids, genome editing, quantitative trait loci (QTLs)

## Abstract

Upland cotton (*Gossypium hirsutum*) is the world’s leading fiber crop and one of the most important oilseed crops. Genetic improvement of cotton has primarily focused on fiber yield and quality. However, there is an increased interest and demand for enhanced cottonseed traits, including protein, oil, fatty acids, and amino acids for broad food, feed and biofuel applications. As a byproduct of cotton production, cottonseed is an important source of edible oil in many countries and could also be a vital source of protein for human consumption. The focus of cotton breeding on high yield and better fiber quality has substantially reduced the natural genetic variation available for effective cottonseed quality improvement within Upland cotton. However, genetic variation in cottonseed oil and protein content exists within the genus of *Gossypium* and cultivated cotton. A plethora of genes and quantitative trait loci (QTLs) (associated with cottonseed oil, fatty acids, protein and amino acids) have been identified, providing important information for genetic improvement of cottonseed quality. Genetic engineering in cotton through RNA interference and insertions of additional genes of other genetic sources, in addition to the more recent development of genome editing technology has achieved considerable progress in altering the relative levels of protein, oil, fatty acid profile, and amino acids composition in cottonseed for enhanced nutritional value and expanded industrial applications. The objective of this review is to summarize and discuss the cottonseed oil biosynthetic pathway and major genes involved, genetic basis of cottonseed oil and protein content, genetic engineering, genome editing through CRISPR/Cas9, and QTLs associated with quantity and quality enhancement of cottonseed oil and protein.

## Introduction

Upland cotton (*Gossypium hirsutum* L.) is the world’s leading fiber crop, as well as one of the most important oilseed crops along with soybean, rapeseed, sunflower and peanut.^1^ The production of the cotton fiber and cottonseed is normally at the ratio of 1:1.65, and cottonseed oil accounts for about 20% of the whole seed weight, and the oil is the second most valuable component of the cotton crop behind fiber, on a price per unit weight basis (O’Brien et al., 2005). Because of its rather neutral flavor, cottonseed oil is commonly desired by the food industry as it does not mask the natural flavor of the food used to cook or process. Cottonseed is also rich in high quality protein containing amino acids that are important for both human consumption (if the toxic gossypol is removed) and animal feeds, especially farm raised fish. The global production of cottonseed protein is estimated to be about 11 million metric tons annually. In fact, cottonseed is the second most important potential source of plant proteins after soybean (Spadaro and Gardner, 1979). However, cottonseed and its derivative products are traditionally regarded as a by-product of the more valuable cotton fiber production, providing only about 14–19% of farm-gate value in cotton production.^2^ The fact that cottonseed is a by-product of cotton production greatly improves its sustainability metrics compared to other oilseeds. Cotton research has thus far been understandably focused primarily on the yield and quality of cotton fiber, while the seed traits, except for seed germination and seed size, are relatively neglected. Consequently, the research and development focus on cottonseeds has been lagging behind other oilseed crops in spite of its abundance in availability and excellent potentials for improvement.

There is a long history of cottonseed oil utilization going back more than 100 years. This arose along with the cotton plantation in the new world and cottonseed oil dominated the vegetable oil market until the rise of soybean oil and canola oil in the 1950s. As Upland cotton production was expanded from the United States to other countries, the use of cottonseed oil for food and protein for animal feed became common in all the cotton growing areas in the world. Cottonseed oil is generally favored due to not only its ready availability and specifically developed extraction technology, but also its bland flavor, does not mask the true flavor of the food that it cooks. Its high smoke point makes it ideal and somewhat superior to other vegetable oils and animal fats for frying applications. As it contains a relatively high level of saturated fatty acids that confers high oxidative stability and high melting point, cottonseed oil has also commonly be used in food industry as “an invisible oil” in the processed snack foods, margarine making and various confectionery applications (Liu et al., 2008; Liu, 2011). More recently, the use of cottonseed oil for renewal fuels (mostly biodiesel) has also attracted considerable attention, as it has a negative carbon profile and could significantly reduce CO_2_ emission in comparison to fossil fuels (Karaosmanoglu et al., 1999; Meneghetti et al., 2007). The whole cottonseed or the meal following oil extraction is rich in proteins and used as popular source of animal feed. Globally, approximately 10 million metric tons of protein is produced by cottonseed (Kumar et al., 2021). Cottonseed protein is endowed with a high level of arginine relative to most plant-based proteins, which has been shown to slow down cancer progression, to act a principal regulator of blood pressure, and to cause a relaxation of cardiovascular smooth muscle cells following conversion to nitric oxide (Lowell et al., 1990; Moncada and Higgs, 1993). Lysine is an important amino acid for humans and animals; and cottonseed kernels contain on average 2.3% lysine (dry weight of kernel powder basis), higher than rice (2.15%) and lower than wheat (2.7%) (Chen et al., 1986). There is an increasing trend of using whole intact cottonseed for feeding lactating dairy cows by leveraging the rumen bypass effects offered by the thick seed coat and remaining fuzz (i.e., linters) following ginning. In addition, cotton is also rich in antioxidants such tocopherols with vitamin E as its main form (Smith and Creelman, 2001).

Despite the continued research focus on cotton fiber, the prospects of increased utilization of cottonseed oil as food, feed and biofuels, have encouraged researchers to develop ways to genetically improve cottonseed products and maximize the outcome for enhanced fiber production and quality, improved nutritional value and expanded industrial applications. Furthermore, there is an environmental impetus to develop such a sustainable byproduct of a valuable fiber crop because of its abundant availability without the need for additional land use and detrimental greenhouse gas emission (Zucker and Zucker, 1943; Ory and Flick, 1994; Alford et al., 1996).

Cotton has a complex genetic base as an allotetraploid species and complicated genetic mechanisms underpinning the accumulation of various valuable metabolites in cottonseed and the development of fibers which cover the seeds. Nevertheless, considerable progress has been made to elucidate the molecular and biochemical mechanism, which has also been used in numerous attempts in genetically enhancing the accumulation or alteration of the relative levels of protein, amino acids, oil, and fatty acid composition in cottonseed. The objective of this review is to summarize and discuss the cottonseed oil biosynthetic pathways and major genes involved, genetic basis of cottonseed oil and protein content, genetic engineering, genome editing through CRISPR/Cas9, and QTLs associated with quantity and quality enhancement of cottonseed oil and protein.

## Cottonseed Oil and Storage Proteins Biosynthesis and Accumulation

**FIGURE 1:**
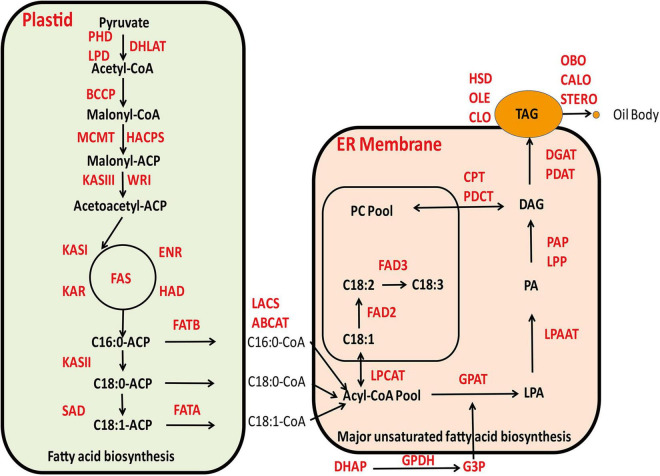
The current understanding for the general biosynthetic pathway of cottonseed oil.

The biochemical processes involved in the biosynthesis of seed oil are relatively well known (Browse and Ohlrogge, 1995; Ohlrogge and Jaworski, 1997; Voelker and Kinney, 2001). Currently, there is a profound understanding on the biochemical and molecular functions of most steps in the lipid biosynthetic pathway, as well as the inheritance of phenotypic performance of various mutants corresponding to these metabolic steps in model plants (Byrne et al., 1996; McMullen et al., 1998). With the advent of numerous high quality genome sequence databases derived from cotton and associated *Gossypium* species, attempts have been made to identify key genes and their interactive gene networks that are involved in oil biosynthesis in cotton (Liu et al., 2009; Jiao et al., 2013; Hovav et al., 2015; Hu et al., 2016; Xu et al., 2016; Zhao et al., 2018c; Ma et al., 2021; Zhu et al., 2021). The current understanding for the general biosynthetic pathway of cottonseed oil is shown in Figure 1.

The major constituent of cottonseed oil is triacylglycerols (TAG) that is comprised of three fatty acids esterified on a glycerol backbone. Cottonseed oil accumulates during the maturation phase of the embryo, which is a highly compartmentalized process including *de novo* biosynthesis of fatty acids mainly occurring in plastids, production of glycerol 3-phoshpate (G-3-P) in cytoplasm and TAG assembly by dehydration condensation of acyl-CoA and G-3-P in endoplasmic reticulum (ER) (Nikolau et al., 2003). The TAG molecules contain the same acyl groups that are also found in membrane lipids, which are predominantly linoleate (18:2), followed by palmitate (16:0), oleate (18:1), stearate (18:0), and linolenate (18:3), in addition to a number of minor fatty acids (Cherry, 1983). The final step of TAG assembly, the acylation of the *sn*-3 position of 1,2 diacylglycerol catalyzed by diacylglycerol acyltransferase (DGAT) to form TAG is commonly regarded as a rate-limiting step (Guo et al., 2017) and plays a substantial role in determining oil content in cottonseed. The resulting TAG molecules in ER will accumulate within a sphere structure known as lipid droplets or oil bodies covered by a monolayer of phospholipids membrane that is decorated with numerous lipid droplets associated proteins, such as oleosin, caleosin, stereoleosin and others. When a lipid droplet reaches a certain size, it will bud off and be released into the cytoplasm (Voelker and Kinney, 2001). It has been generally recognized that *DGAT1* plays a crucial role in determining TAG production as it catalyzes the rate-limiting step to convert DAG into TAG. However, there are reports that *DGAT3* may play a more active role in promoting TAG biosynthesis in light of transcriptome analysis of developing cottonseeds, although empirical evidence is required (Hovav et al., 2015; Zhao et al., 2018c). A considerable body of literature is now available to imply that a number of transcription factors, such as *WRI1*, are playing an imperative role in fostering the carbon reallocation toward to *de novo* fatty acids biosynthesis in plastids and rendering increased availability for TAG biosynthesis (Kong et al., 2019). In congruence, *WRI1* and *NF-YB6* were highly expressed and displayed coordinated temporal patterns with oil accumulation in cottonseeds (Zhao et al., 2018b). That the key genes involved in *de novo* fatty acid biosynthesis, such as *SAD6* and *FATA*, showed clear differential expression concomitant with oil accumulation, which were substantially highly expressed in *G. barbadense* than in *G. hirsutum*, highlighting the divergence between these two closely related allotetraploid cottons (Zhu et al., 2021). Some other enzymes in plastids, such as *GhPEPC1*, are not only involved in photosynthesis but also key to the inflowing of carbon turnover to fatty acid biosynthesis attributable to the accumulation of cottonseed oil (Xu et al., 2016). In higher plants, seed storage proteins are synthesized on the rough ER, using amino acids directly taken up by the embryo, or obtained after transamination reactions. Subsequently, they are transported into protein storage vacuoles by a vesicle-mediated pathway (Jolliffe et al., 2005). In cottonseed, two major classes of storage proteins are globulins and albumins, which differ in their solubility properties. Both globulins and albumins are synthesized and compartmentalized in storage protein vacuoles during cottonseed maturation (Dure and Chlan, 1981).

## Factors Affecting Cottonseed Oil and Protein Contents

The contents of oil and protein in cottonseeds are quantitative traits that are simultaneously affected by genetic and environmental factors and their interactions. Cottonseed oil and protein contents often vary among different growing seasons, growing locations and years (Singh et al., 1985; Dani and Kohel, 1989; Ye et al., 2003). The interactive effects of the genotype × environment depend on not only environmental factors such as water, fertilizer, soil, light and temperature, but also on the relative contribution of the parent genotypes to the trait (Wang, 1992; Hom et al., 2015). However, the fatty acid composition of seed oil is mainly determined by the genotype of the developing embryo (embryogenic control) (Downey and Harvey, 1963; Ecker and Yaniv, 1993; Velasco et al., 2004). Kohel (1980) estimated a moderate heritability based on a 20 × 5 NCII design and a low heritability based on F_2_/F_3_ regression for cottonseed oil content. Heritability estimates for oil content varied from low (Meredith et al., 2012) to moderate (Zeng et al., 2015; Campbell et al., 2016; Kothari et al., 2016) or high (Yu et al., 2012; Zhao et al., 2019), depending on different genetic backgrounds of cultivars and testing environmental conditions in these studies. Singh et al. (1985) showed that the contents of protein and oil exhibited non-additive genetic effects with a substantial environmental influence based on a set of diallel crosses involving ten parents. However, Yuan et al. (2001) showed that oil content was controlled mainly by maternal additive effect, while protein content by direct additive effect in F3 seeds harvested from F2 hybrids between four Upland lines with the double recessive (*gl_2_gl_3_*) glandless trait and five Upland lines with the dominant glandless (*Gl_2_*^e^**) trait. Based on F3 hybrids between 13 cotton chromosome substitution lines (CSLs) each carrying a pair of chromosomes or arms from *G. barbadense* and five elite Upland cultivars, Wu et al. (2010) confirmed that seed oil content had significant cytoplasmic effects and also dominance effects, while protein content had significant embryo additive effects based on the additive and dominance (AD) genetic model with cytoplasmic effects. In 316 Upland cotton accessions genotyped by 390 K SNPs, Du et al. (2018b) indicated that cottonseed protein, oil, palmitic, linoleic, oleic, myristic and stearic acid contents exhibited significant additive and dominance effects; however, the epistatic effects and genotype-environment interactions were largely diverse across traits. Therefore, cottonseed oil and protein contents are heritable traits that are controlled by multiple genes with additive and dominant effects with variable heritability estimates.

The content of cottonseed oil is strongly and negatively correlated with protein content (Hanny et al., 1978; Kohel and Cherry, 1983; Wu et al., 2009; Yu et al., 2012; Hinze et al., 2015; Campbell et al., 2016), but positively correlated with fiber length, fiber uniformity and fiber strength (Kothari et al., 2016). A recent study by Yuan et al. (2019) confirmed the significant positive correlation of the total fatty acid content in cottonseeds with fiber length and strength and its significant negative correlation with fiber uniformity. Furthermore, palmitic acid content was significantly and positively correlated with fiber elongation. However, the study further showed that the reverse was true for the correlations of cottonseed protein content with fiber length, strength, and uniformity. The correlation analysis further suggested that the above well-documented negative association between seed protein and oil contents may be to some extent attributed to the negative correlation between oleic acid and protein content. Research efforts to increase the oil content of cottonseed, which will most likely also decrease the level of protein, will actually have a positive impact on cottonseed value and sustainability metrics due to being able to reduce the level of fertilizer (mainly supplemental nitrogen) applied to the plant to support protein production in the seed. Increasing seed oil and reducing seed protein will therefore positively impact the carbon footprint for cotton production and utilization.

## Genetic Variation Within *Gossypium* and Classical Genetic Studies of Cottonseed Oil, Fatty Acid, and Protein Contents

One of the biggest challenges in improving the cottonseed quality traits is the limited amendable genetic variability within cotton germplasm, despite the existence of great genetic variation in cottonseed oil content (17–27%) and protein content (16–36%) among cotton species and cultivars (Kohel, 1980; Wu et al., 2009; Dowd et al., 2010; Kothari et al., 2016). Sharif et al. (2019) showed that oil content was the highest in *G. lobatum* (24.82%) and *G. harknessii* (24.22%), whereas the Old World wild species had lower oil contents including *G. stocksii* and *G. somalense* with the lowest oil content (11.22%). Among the four cultivated species, *G. barbadense* had the highest oil content, followed by *G. hirsutum*; and the two A-genome diploid species (*G. herbaceum* and *G. arboreum*) showed the lowest level. After comparing 33 *Gossypium* species, Hinze et al. (2015) confirmed that diploid species except for the A- and K- genome species possessed the lowest oil and protein contents. Tetraploids (21.7%) and the K-genome species (21.4%) had the highest oil content (21.7%). In addition, large ranges in oil contents within the D genome and each cultivated species were observed. Agarwal et al. (2003) and Khan et al. (2015) also found significant variability for oil content in the cotton germplasm collections in India and Pakistan, respectively. Therefore, sufficient genetic variability in cottonseed oil exists within the *Gossypium* genus which could be utilized for making genetic gains (Kohel, 1978; Horn et al., 2011). However, the genetic improvement in oil accumulation of cottonseed is constrained by the rather limited variation among the elite Upland cotton (*G. hirsutum*) cultivars and lines as the result of extensive selection within the species toward improving lint yield and quality. Significant variation in cottonseed oil and protein contents may exist between cultivars and race stocks within Upland cotton. Hinze et al. (2015) showed that cultivated tetraploid accessions had higher oil content (22.7%) than wild tetraploid accessions (20.9%). Among four Upland cotton breeding lines and four semi-wild *G. hirsutum* accessions tested in multi-environments, Kothari et al. (2016) showed that the oil content ranged from 13 to 27% and the protein values ranged from 16 to 36%. The variability for oil content in elite cultivars and lines of *G. hirsutum* ranged from 14.5 to 22.0% with mean of 19.2% (Pandey, 1977). A classic breeding approach through crosses between selected germplasm led to a moderate increase in oil content (21.20–26.30%) as compared to their parents (21.48–24.16%) (Dani, 1988). To overcome the bottleneck of low genetic variation in existing Upland cotton, the cultivated allotetraploid cotton can be crossed with diploid cottons, followed by selection to improve oil accumulation (Thiagarajan and Ramaswamy, 1982). In addition to oil content, natural variations in fatty acid components such as oleic acid, myristic acid, linoleic acid and linolenic acid in cottonseed oil, have also been reported (Lukonge et al., 2007). However, fatty acid profiles in large cotton germplasm collections and breeding populations remain to be analyzed.

As for cottonseed protein, based on results from a large cotton germplasm collection (1,335 and 1,234 accessions in the Mississippi and Texas location, respectively), Kohel et al. (1985) suggested that there was sufficient variability for genetic improvement. Hinze et al. (2015) detected a wide range of protein content (10–36%) and oil content (8–27%) in 2,256 accessions representing five tetraploid and 28 diploid *Gossypium* species, and the results showed that wild diploid species generally had extremely low cottonseed protein contents. The Old World A-genome species had the highest protein content (23.8%), followed by the tetraploid species (21.6%). Cultivated tetraploid accessions had a wider range (14.9–35.9%) protein content than wild tetraploid accessions (15.4–30.7%) although both groups had similar mean protein content. Variation in protein components and relative content of the protein subunits were also investigated among cultivars (Song and Zhang, 2007).

In general, considerable variation in oil content and proteins content have been identified in cottonseed; however, inconsistency and relatively small magnitude of the variations poses significant challenge for their utilization in cotton breeding programs. Nonetheless, the existence of the variation offers promise for the discovery of greater variation if a greater segment of cotton germplasm is explored, especially the wild *Gossypium* species and those beyond the mainstream germplasm collections such as exotic cotton germplasm.

## Quantitative Trait Loci Mapping and Genomewide Association Studies of Cottonseed Oil, Fatty Acid, and Protein Contents

Using linkage mapping and genomewide association studies (GWAS), QTLs associated with contents of cottonseed oil, fatty acids, protein, and amino acids have been detected in specifically designed genetic populations Zhang et al., 2022. Song and Zhang (2007) were among the first to report a single QTL for kernel oil percentage in an interspecific BC_1_S_1_ population derived from an interspecific *G. hirsutum* (Gh) TM-1 × *G. barbadense* (Gb) Hai 7124 cross based on simple sequence repeat (SSR) markers. This was followed by Yu et al. (2012) who mapped 12 QTLs in relevance to cottonseed oil, protein and gossypol contents using a different interspecific population comprised of backcross inbred lines (BILs). Up to date, a total of more than 160 QTLs for cottonseed oil content and more than 130 QTLs for different fatty acids were identified from at least 14 published studies (Table 1). These mapping populations included three Gh × Gb populations and five recombinant inbred lines (RIL) populations for linkage mapping, and six accession panels for GWAS. A meta-analysis was previously performed using the cotton QTL database with only a few reports on cottonseed oil and protein contents (Said et al., 2013, 2015a,b).^3^ More studies have been published since then, which requires a meta-analysis of QTLs to identify consistent QTLs and QTL hotspots or clusters across environments and genetic populations. It appears that some of the QTLs were in common or on similar chromosomal locations between/among studies. As a result, candidate genes for oil QTLs were identified or validated (Ma et al., 2019; Liu et al., 2020; Zhang et al., 2021). Furthermore, QTLs corresponding to various fatty acids were identified in other studies (Liu G.Z. et al., 2015; Du et al., 2018b; Yuan et al., 2018). However, only a small number of these QTLs were identified in multi-environments or multiple genetic backgrounds. For example, Ma et al. (2019) identified 19 QTLs for cottonseed oil content in multiple environments based on GWAS in Upland cotton, and a peroxidase (*PRXR1*) gene was confirmed to be the candidate gene within one of the QTL regions *via* virus induced gene silencing (VIGS). More recently, Zhang et al. (2021) reported that only five of 39 QTLs for cottonseed oil content were stable across different environments in a RIL population of 196 lines, and several genes including one coding for a transcription factor within the stable QTL regions were differently expressed during ovule development. Till now, none of the QTLs reported have been tracked using associated markers in genetic populations for cottonseed oil improvement. Hence, their direct application in marker-assisted selection (MAS) for oil content and quality is still unknown.

**TABLE 1:** Quantitative trait loci (QTLs) mapped for cottonseed oil content and fatty acids.

Authors	Year	Mapping population	Traits	Markers	No. QTL	QTL details
Liu H.Y. et al. (2017)	2017	188 Gh × Gh RILs	Amino acids	SSRs, SRAPs, RAPDs	56	On c3, c5, c6, c9, c16, c18, c21, c22, c25, c23, LG3, LG4, LG5, LG6, LG8, LG10, LG11
Liu et al. (2012)	2012	376 lines Gh × Gh F_2_	Protein contents		12	On c22, c25, c5, LG3, LG5, LG6
Liu et al. (2013)	2013	188 Gh × Gh RILs	Amino acids	SSRs, SRAPs, RAPDs	35	A5, A6, A8, D15, D18, D22, D23, LG5, LG6, LG7, LG11, LG12
Song and Zhang (2007)	2007	140 Gh × Gb BC1S1	Amino acids, oil, protein	SSRs	8,1	On D8
Yu et al. (2012)	2012	146 Gh × Gb BILs	oil, protein	392 SSRs	17,22	On c12
Alfred et al. (2012)	2012	376 IF2 from 188 RILs, Gh	oil	388 SSRs	4	c18 (2), c22 (1) and LG 11 (1)
Liu G.Z. et al. (2015)	2015	180 Accessions, Gh	Oil, protein	228 SSRs	15	15 SSRs on A3, A7, A9, A10, A12, A13, D2, D5, D6, and D9
Liu D.X. et al. (2015)	2015	270 RILs, Gh	Oil, protein, fatty acids	1,675 SSRs	15	15 crude oil, 8 linoleic, 10 oleic, 13 palmitic and 12 stearic acid QTL
Badigannavar and Myers (2015)	2015	75 Elite lines, Gh	Oil, protein	234 AFLP	6	Chromosomes undetermined
Shang et al. (2016)	2016	2 RIL pop and 2 BC pop, Gh	Fatty acids	1,053 SSRs	24	On 13 chromosomes
Zeng et al. (2016)	2016	277 Accessions, Gh	Oil, protein	24 SNPs	2	One main-effect QTN, one epistatic QTN
Du et al. (2018b)	2018b	316 accessions, Gh	Oil, fatty acids, protein	390,000 SNPs		16 Protein, 21 oil and 87 fatty acids (palmitic, linoleic, oleic, myristic and stearic)
Yuan et al. (2018)	2018	196 Accessions, Gh	Oil, protein, fatty acids	41,815 SNPs	28	6 Protein, 2 myristic, 4 oleic, 8 stearic, 4 palmitic, 4 linoleic and 8 oil content QTL
Wang et al. (2019)	2019	180 RILs, Gh	Oil, protein	7,033 SLAF-SNPs	17	On c1, c3, c5, c12 (2), c15, c16, c19 (3), c20, c21 (3), c24, and c25
Zhao et al. (2019)	2019	503 Accessions, Gh	Oil	179 SSRs/11,975 SNPs	8	On c1, c10, c12, c13, c15, c17, c24
Ma et al. (2019)	2019	90 + accessions, Gh	Oil	15,369 SNPs	13	On 13 chromosomes including 1 on D05 with a candidate gene
Liu et al. (2020)	2020	376 IF2 from 188 RILs, Gh	Oil	388 SSRs	8	Including a QTL on A02 with 2 candidate genes
Zhu et al. (2020)	2020	325 CSSLs, Gh × Gb	Oil	11,653,661 SNPs	15	On A01 (4), A03, A05, A07, A11, A12 (2), A01 (2), A03 (2), and A10
Zhang et al. (2022)	2022	188 Gh × Gh RILs	Oil, fatty acids	388 SSRs	15	On c3(1), c18(4), lg3(2), lg7, lg8, c15(1), lg6(3), c16(1), c15(1)
Zhang et al. (2021)	2021	196 Gh × Gh RILs	Oil	8, 295	39	c1(1), c3(1), c4(5), c5(2), c6(1), c7(2), c9(1), c10(3), c11(1), c12(2), c13(3), c14(4), c15(3), c17(1), c19(1), c20(1), c22(1), c24(8)

Chromosomes or genes introduced to *G. hirsutum* from *G. barbadense* and other tetraploid *Gossypium* species were found to affect cottonseed oil content substantially, based on CSLs (Wu et al., 2009, 2010; Bellaloui et al., 2020; Saha et al., 2020). Multiple QTL alleles from *G. barbadense* were demonstrated as highly promising for enhancing seed oil content in introgressed *G. hirsutum* lines (Zhu et al., 2020). Therefore, introgression breeding between *G. hirsutum* and *G. barbadense* may greatly improve the content of seed oil content and possibly fatty acid composition. These QTLs detected for seed quality traits in cotton are expected to be useful in cotton breeding to develop cotton with improved cottonseed nutrient quality. However, to date, all the genetic populations developed were small in size (100–200 progeny), which limited genetic recombination between parents. The extent to which cottonseed oil content can be increased and oil quality can be enhanced as the result of alteration in fatty acid composition through extensive introgression breeding remains unknown. The goal of research and breeding effects is to transfer the identified desirable QTLs into elite cotton cultivars for the improvements of both oil accumulation and oil quality without trade-offs in fiber yield or quality.

## Gene Expression Studies During Cottonseed Oil and Protein Accumulation

Lipids and fatty acids are a large class of compounds existing in plants, and most edible vegetable oil consists of a few common fatty acids, including saturated, monounsaturated, and polyunsaturated fatty acids. Fatty acids are stored in seeds in the form of triacylglycerol (TAG). Therefore, the TAG biosynthetic pathway involving many enzymes has become one of the hallmarks of lipid biochemistry. Cottonseed oil contains 71% unsaturated fatty acids and 28% saturated fatty acids. Unsaturated fatty acids include 58% linoleic acid (18:2) and 13% oleic acid (18:1), and saturated fatty acids include 26% palmitic acid (16:0) and 2% stearic acid (18:0) (Cherry, 1983). In addition, there are many other minor fatty acids including dihydrosterculic acid (DHSA) (Dowd et al., 2010; Dowd, 2012).

Since the lipid and protein biosynthetic pathways compete for the same substrate using phosphoenolpyruvate through acetyl-CoA carboxylase (ACCase) and phosphoenolpyruvate carboxylase (PEPC), respectively, it is not surprising that cottonseed oil and protein contents are negatively correlated as reported previously (Hanny et al., 1978; Kohel and Cherry, 1983; Wu et al., 2009; Yu et al., 2012; Hinze et al., 2015; Campbell et al., 2016). Cui et al. (2017) showed that overexpression of *GhACCase* subunits resulted in increased cottonseed oil content by 17–22%. A large number of oil-related genes, such as *fatty acyl-ACP thioesterase B* (*FATB*), *acyl carrier protein 5* (*ACP5*) (Yuan et al., 2018) and *KASIII* (Du et al., 2018a), have been identified by various approaches including GWAS (e.g., Du et al., 2018a,b), gene expression studies (e.g., Ma et al., 2021; Zhu et al., 2021), cloning and sequence-based *in silico* analysis (Zhang et al., 2009; Yu et al., 2011; Yurchenko et al., 2014; Shang et al., 2016; Cui et al., 2020). Among *GhSAD* genes coding for stearoyl-acyl carrier protein desaturase, *GhSAD4* was found to stand out as the most relevant to determine the relative ratio of oleic acid and linoleic acid (Shang et al., 2017). Of 17 *SAD* gene family members identified in Upland cotton, *GhA-SAD6* and *GhD-SAD8* have strong substrate specificity for 16:0-ACP, and *GhA-SAD5* and *GhA-SAD7* exhibited a high specific activity on 18:0-ACP (Liu et al., 2019). Tetraploid cotton genomes contain 13 *LPAAT* genes, including five on Dt subgenome and eight on the At subgenome (Wang et al., 2017). Based on a further sequence variation and gene expression analysis, genetic modification to overexpress single genes like *At-Gh13LPAAT5* was found to be effective in improving the production of total TAG and oil content (Wang et al., 2017). In addition to these genes that have been well known for their role in fatty acid biosynthesis, other genes that encode less studied proteins, such as a calcium-dependent lipid-binding (*CaLB*) protein (Zhao et al., 2019) and a peroxidase (*PRXR1*) (Ma et al., 2019) were also implicated for their roles in determining cottonseed oil content based on GWAS followed by confirmation using VIGS.

## Genetic Engineering of Cotton for Improving Cottonseed Oil, Fatty Acid, and Protein Contents

While the focus of cotton breeding on improving fiber quality will not change, there is an increased interest in enhancing the value of cottonseed by enhancing seed oil production and improving the nutritional and functional properties of the cottonseed oil (Liu et al., 2009). In the earliest attempts to genetically improve cottonseed oil, modest changes in oil content and fatty acid composition were achieved in Acala cotton through traditional breeding (Cherry et al., 1981; Cherry, 1983), reflecting the meager genetic variation available in natural germplasm and elite breeding lines. However, the improvements in molecular mechanisms underpinning the genetic variation and biochemical pathways, as well as the advent of genetic engineering approaches provide an alternative to rapidly alter carbon metabolism and manipulate lipid composition in cottonseed. Genetic modification of cottonseed oil has also been made more efficient through a series of methodological advancements in transgene expression systems, plant regeneration from tissue culture and gene transformation *via Agrobacterium tumefaciens* or particle bombardment (Zhang, 2015).

RNA interference (RNAi) attenuations in the expressions of genes coding for fatty acid desaturase (*FAD2*) on chromosome D12, stearoyl-ACP desaturase 1 (*SAD1*) on D9 and β-ketoacyl-acyl carrier protein synthase (*KASII*) in cotton resulted in substantially altered fatty acid composition, with particularly enhanced levels in oleic acid (from 13 to 78%), stearic acid (from 2 to 40%) and palmitic acid (from 26 to 15%), respectively, in cottonseed (Liu Q. et al., 2002, 2017). In addition, RNAi-directed down-regulation of *PEPC2* up-regulated most lipid synthesis-related genes, resulting in 7.3% increase in cottonseed oil content (Zhao et al., 2018a). Shockey et al. (2017) and Sturtevant et al. (2017) identified a natural mutant allele of *FAD2-1D* in *G. barbadense* with high oleic acid in cottonseed oil, and its incorporation into *G. hirsutum* doubled oleic acid content (Dowd et al., 2020). Most recently, Chen et al. (2021) confirmed that knockout mutants of the *GhFAD2* genes in Upland cotton by CRISPR/Cas9 editing increased the oleic acid level to 77.7% with a concomitant decrease in linoleic acid (from 58.6 to 6.9%) and palmitic acid (from 23.95 to 13.18%). Transforming Upland cotton with an *FAD3* gene from *Brassica napus* and a D6D gene from *Echium plantagineum* resulted in approximately 30% α-linolenic acid (ALA) and 20% γ-linolenic acid (GLA), respectively, with no change in total oil content (Gao et al., 2020). Table 2 presents a summary of the genes that have been genetically engineered to improve cottonseed oil content and fatty acid composition.

**TABLE 2:** Genes used in genetically engineering cotton for improvement of cottonseed oil.

Gene	Transgenic event	Major results	Authors
*GhPEPC1*	RNAi,Gh	Up to 16.7% increase in oil content	Xu et al., 2016
*GhPEPEC2*	RNAi, Gh	Seed oil increased by 7.3%	Zhao et al., 2018a
*Ghfad2*	Suppression,Gh	Oleic acid increased from 15 to 21–30%	Chapman et al., 2001
*BnFAD2*	Non-functional,Gh	Oil content reduced from 20 to 12%	Chapman et al., 2008
*ghFAD2-1*	RNAi,Gh	Oleic acid increased from 13 to 78%	Liu Q. et al., 2002
*BnFAD3*	Insertion,Gh	30% Alpha-linolenic acid(ALA)	Gao et al., 2020
*ghSAD-1*	RNAi, Gh	Stearic acid increased from 2 to 40%	Liu Q. et al., 2002
*GhACCase*	Overexpression, Gh	17–21% increase in cottonseed oil content	Cui et al., 2017
*GhFATB GhFAD2-1*	RNAi, Gh	Increase oleic acid content by 156.96%, decreased palmitic acid content by 21.28%, decreased linoleic acid by 33.92%	Liu F. et al., 2017
*GhKASII*	RNAi, Gh	Palmatic acid increased from 25 to 51%	Liu Q. et al., 2017
*GhPRXR1*	VIGS,Gh	Cottonseed oil content decreased by18%	Ma et al., 2019
*Gh13LPAAT5*	Transformed yeast	25–31% Increase in palmitic and oleic acid	Wang et al., 2017
		16–29% Increase in tricacylglycerol(ATG)	
*GhWRII-7*	Mutant Arabidopsis	Lipid content reduced by 3times	Zang et al., 2018
*GhWRI1*	Arabidopsis	Increased from 19.85 to 25.25% in cottonseed oil content	Zhao et al., 2018b
*GhCIPK6*	Insertion, Gh	Oil content reduced to 25.4–32.7% from 33.6%	Cui et al., 2020
*GhDGAT1*	Overexpression, Gh	4.7–13.9% Increase in cottonseed oil content	Wu et al., 2021
*GhDOfI*	Overexpression,Gh	Increased oil content	Su et al., 2017
*GhA-SAD6,GhD-SAD8*	Overexpression,Gh	Increase of palmitoleic acid by at least 4–5 folds.	Liu et al., 2019
Δ*6desaturase*	Insertion, Gh	y-linolenic acid	Gao et al., 2020
*GhFAD2-1A/D*	CRISPR/Cas9,Gh	Up to 77.72% increase in oleic acid and decreased concomitantly from 58.62 to 6.85% in linoleic acid	Chen et al., 2021

Cottonseed oil is featured with a small amount of rare cyclic fatty acids, including DHSA and its downstream products sterculic acid and malvalic acid, all of which have been found to suppress mammalian Stearoyl-CoA desaturase activity and improve liver metabolomic profiles in high fat fed mice (Paton et al., 2017). The key genes encoding for cyclopropane fatty acid synthases converting oleic acid to DHSA have been identified in *Arabidopsis* (Bao et al., 2002) and cotton (Yu et al., 2011). Although the proof of concept has been made in producing DHSA in transgenic model plants (Yu et al., 2018; Okada et al., 2020), associated genes have yet to be modified in the cotton genome to raise DHSA production in cottonseeds.

It should be recognized that a radical modification of fatty acid composition may have deleterious effects on membrane integrity and impede seed germination under conventional farming practice, even though the modification was transcriptionally controlled by seed-specific promoters. For example, in the case that a leaky “seed-specific” promoter was used, severe compromises in plant growth and development, especially under environmental stresses, and penalty in yield may occur as a result (Lindgren et al., 2003). Further, the commercial planting of genetically modified crops generated by transgenic approaches especially those by agrobacterium mediated transformation assisted by selectable markers such as kanamycin resistance, has met substantial public skepticism and resistance in addition to lengthy and heavy regulatory burdens (Shockey et al., 2017). However, the recent availability of versatile genome editing techniques, such as transcription activator-like effector nucleases (TALEN) and clustered regulatory interspaced short palindromic repeats (CRISPR)/Cas9 systems has allowed scientists to precisely edit the expression of target genes without T-DNA insertion. Most importantly, the use of genome editing techniques may circumvent the lengthy regulatory processes and renders its products for rapid commercialization (Wang et al., 2020; Zhang et al., 2020).

Although cottonseed storage proteins are generally deficient in essential amino acids, especially lysine, which can be inadequate from a nutrition point of view, synthetic forms of lysine and other essential amino acids can be added to the diet to correct the deficiency. Cottonseed protein also tends to be deficient in isoleucine and the sulfur-rich amino acids such as methionine and cysteine (Capdevila and Dure, 1977). The sulfur-rich proteins, such as albumin, constitute a low fraction of the total cottonseed proteins (Galau et al., 1992; Hu et al., 2011). Genetic improvement of cottonseed storage protein and amino acid profiles is clearly long overdue, which could be developed in concert with the development of gossypol-free trait to meet the nutritional requirement for use as a source of high quality plant protein for non-ruminant animals or humans. The broad application of cottonseeds for human consumption and as animal feed is considerably constrained by the presence of gossypol which is sequestered in the pigment glands of cottonseed and other plant tissues. Gossypol is in a class of polyphenol compounds (terpenoids) that can be toxic and nutritionally undesirable, if safe levels in the diet are exceeded. Natural glandless (devoid of gossypol) cotton mutants exist in cotton and have been extensively studied and used in breeding (Zhang and Wedegaertner, 2021). Genetic modified glandless cottonseed has also been developed by RNAi down-regulation of cadinene synthase (Sunilkumar et al., 2006), which is currently being incorporated into elite Upland cotton cultivars to enable broad applications of cotton proteins for human consumption and monogastric animals. Commercialization of this technology has been slow due to international regulatory hurdles for genetically modified crops. Most recent reviews on genetics, breeding and genetic engineering to develop glandless cotton can be found in Rathore et al. (2020) and Zhang and Wedegaertner (2021).

Natural genetic variation in vitamin E also exists within cotton (Smith and Creelman, 2001). For example, several long-staple Acala 1517 cultivars were higher in α-tocopherol than medium-staple Upland cultivars. However, the genetic and genomic basis of the variation is currently not understood. Radcliffe and Czajka-Narins (2006) showed that cottonseed oil had a lowering effect on total cholesterol content for both male and female rats, but on high-density lipoprotein cholesterol for male rats only, and the replacement of corn oil with cottonseed oil resulted in changes in tocopherol status. A follow-up study in human by Radcliffe et al. (2009) further showed that cottonseed oil used in muffins and potato chips even increased vitamin E intake. Recently, Salimath et al. (2021) reported that genetic modification by converting tocopherols into more potent form of tocotrienols *via* introducing homogentisate geranylgeranyl transgenic coding sequence under the control of the *Brassica napus* seed-specific promoter from barley through genetic engineering. Transgenic cottonseeds had a 2–3-fold increase in the accumulation of total vitamin E (tocopherols + tocotrienols), with more than 60% γ-tocotrienol.

## Prospective

As a byproduct in cotton production, cottonseed has excellent potential for use as a source of sustainable, high quality vegetable oil, biofuel and proteins because of its abundance that is expected to grow as the demand for cotton fiber continues to increase. Genetic improvements in nutritional value and functional properties of cottonseeds are being leveraged by the rapid advancements in biotechnology and genomics-based molecular breeding. In this review, we have summarized the most recent advances in genetic improvement of cottonseeds in relevance to the content of oil or protein, fatty acid composition that have been demonstrated to be amendable. Genetic improvements of cottonseed traits have proven to be particularly challenging as cottonseed is relatively low value product compared to cotton fiber that commends more than 85% of the farm-gate value of cotton production. This necessitates the employment of high precision genome editing technology and molecular breeding strategies to enable achieving genetic improvements in seed traits without trade-offs in fiber production and quality, as well as regulatory hurdles. Although cotton is among the earliest crops being grown commercially, the path leading to a successful commercialization and public acceptance of genetic modified cottonseed oils or whole seeds as a novel source of food grade proteins with improved nutritional value may not be an easier task in comparison to other genetic modified non-food crops. Nevertheless, more and more proof of concept studies have been conducted in model plants and recently in cotton that renders cotton industry standing on a new threshold of research and development, equipped with ever increasing knowledge in the intricate relationships and carbon reallocation between seed and fiber, and new sets of tools with high precision for modifying the cotton genome. It could be envisioned that the development of nutritionally improved and functionally versatile cottonseed, perhaps led by the development of high oleic cottonseed oil that could emulate the success of high oleic soybean oils, such as Plenish, Vistive Gold, and Calyxt, will come to fore, along with the continuous and synchronized development in cotton fiber.

## Author Contributions

MW finalized the summary of all the publications used in this review and wrote the manuscript. JY directed the study and contributed to the writing of the manuscript. JZ provided Tables 1, 2 and an early incomplete draft of the manuscript, and finalized the manuscript. TW edited the manuscript. WP contributed to the writing of the manuscript. All authors read and approved the final manuscript.

## Conflict of Interest

TW was employed by Cotton Incorporated. The remaining authors declare that the research was conducted in the absence of any commercial or financial relationships that could be construed as a potential conflict of interest.

## Publisher’s Note

All claims expressed in this article are solely those of the authors and do not necessarily represent those of their affiliated organizations, or those of the publisher, the editors and the reviewers. Any product that may be evaluated in this article, or claim that may be made by its manufacturer, is not guaranteed or endorsed by the publisher.

## Footnotes

1
www.statista.com/statistics/267271/worldwide-oilseed-production-since-2008

2
www.cotton.org/econ/cropinfo/costsreturns/usa.cfm

3
www.cottonqtldb.org
